# New Protein-Coated Silver Nanoparticles: Characterization, Antitumor and Amoebicidal Activity, Antiproliferative Selectivity, Genotoxicity, and Biocompatibility Evaluation

**DOI:** 10.3390/pharmaceutics13010065

**Published:** 2021-01-07

**Authors:** Lucía Margarita Valenzuela-Salas, Alberto Blanco-Salazar, Jesús David Perrusquía-Hernández, Mario Nequiz-Avendaño, Paris A. Mier-Maldonado, Balam Ruiz-Ruiz, Verónica Campos-Gallegos, María Evarista Arellano-García, Juan Carlos García-Ramos, Alexey Pestryakov, Luis Jesús Villarreal-Gómez, Yanis Toledano-Magaña, Nina Bogdanchikova

**Affiliations:** 1 Facultad de Ciencias de la Salud, Universidad Autónoma de Baja California, Tijuana 22260, Baja California, Mexico; lucia.valenzuela@uabc.edu.mx (L.M.V.-S.); paris.mier@uabc.edu.mx (P.A.M.-M.); 2 Programa de Maestría y Doctorado en Ciencias e Ingeniería (MyDCI), Facultad de Ciencias, Universidad Autónoma de Baja California, Ensenada 22860, Baja California, Mexico; alberto.blanco.salazar@uabc.edu.mx; 3 Escuela de Ciencias de la Salud Unidad Valle Dorado, Universidad Autónoma de Baja California, Ensenada 22890, Baja California, Mexico; 4 Facultad de Química, Universidad Nacional Autónoma de México, Ciudad de México 04510, Mexico; dperrusquiah@gmail.com; 5 Unidad de Medicina Experimental, Facultad de Medicina, Universidad Nacional Autónoma de México, Ciudad de México 06720, Mexico; manequiz@yahoo.com.mx; 6 Facultad de Medicina unidad los Mochis, Universidad Autónoma de Sinaloa, Los Mochis 81223, Sinaloa, Mexico; balamruiz@uadeo.mx; 7 Facultad de Ciencias, Universidad Autónoma de Baja California, Ensenada 22860, Baja California, Mexico; 8 Departamento de Ciencias de la Salud, Universidad Autónoma de Occidente, Los Mochis 81223, Sinaloa, Mexico; a308236@uabc.edu.mx (V.C.-G.); evarista.arellano@uabc.edu.mx (M.E.A.-G.); 9 Research School of Chemistry and Applied Biomedical Sciences, Tomsk Polytechnic University, 634050 Tomsk, Russia; pestryakov2005@yandex.ru; 10 Facultad de Ciencias de la Ingeniería y Tecnología, Universidad Autónoma de Baja California, Tijuana 22260, Baja California, Mexico; luis.villarreal@uabc.edu.mx; 11 Centro de Nanociencias y Nanotecnología, Universidad Nacional Autónoma de México, Ensenada 22860, Baja California, Mexico; nina@uabc.edu.mx

**Keywords:** non-genotoxic silver nanoparticles, biocompatibility, antitumor activity, human adenocarcinoma HCT-15, amoebicidal activity, *Entamoeba histolytica*, antiproliferative selectivity, medium lethal dose (LD_50_), GHS category 5

## Abstract

Nanomaterials quickly evolve to produce safe and effective biomedical alternatives, mainly silver nanoparticles (AgNPs). The AgNPs’ antibacterial, antiviral, and antitumor properties convert them into a recurrent scaffold to produce new treatment options. This work reported the full characterization of a highly biocompatible protein-coated AgNPs formulation and their selective antitumor and amoebicidal activity. The protein-coated AgNPs formulation exhibits a half-inhibitory concentration (IC_50_) = 19.7 µM (2.3 µg/mL) that is almost 10 times more potent than carboplatin (first-line chemotherapeutic agent) to inhibit the proliferation of the highly aggressive human adenocarcinoma HCT-15. The main death pathway elicited by AgNPs on HCT-15 is apoptosis, which is probably stimulated by reactive oxygen species (ROS) overproduction on mitochondria. A concentration of 111 µM (600 µg/mL) of metallic silver contained in AgNPs produces neither cytotoxic nor genotoxic damage on human peripheral blood lymphocytes. Thus, the AgNPs formulation evaluated in this work improves both the antiproliferative potency on HCT-15 cultures and cytotoxic selectivity ten times more than carboplatin. A similar mechanism is suggested for the antiproliferative activity observed on HM1-IMSS trophozoites (IC_50_ = 69.2 µM; 7.4 µg/mL). There is no change in cell viability on mice primary cultures of brain, liver, spleen, and kidney exposed to an AgNPs concentration range from 5.5 µM to 5.5 mM (0.6 to 600 µg/mL). The lethal dose was determined following the OECD guideline 420 for Acute Oral Toxicity Assay, obtaining an LD_50_ = 2618 mg of Ag/Kg body weight. All mice survived the observational period; the histopathology and biochemical analysis show no differences compared with the negative control group. In summary, all results from toxicological evaluation suggest a Category 5 (practically nontoxic) of the Globally Harmonized System of Classification and Labelling of Chemicals for that protein-coated AgNPs after oral administration for a short period and urge the completion of its preclinical toxicological profile. These findings open new opportunities in the development of selective, safe, and effective AgNPs formulations for the treatment of cancer and parasitic diseases with a significant reduction of side effects.

## 1. Introduction

In the last decades, nanotechnology evolved to improve material properties focused on biomedical applications as antimicrobials [1], antiviral [2], drug delivery systems, treatment of cancer [3], and bioimaging [4], among others [5,6]. Nanomedicine and nanotoxicology are focused on determining the effectiveness and risk of nanomaterials used for specific biomedical and therapeutic applications. However, the lack of systematicity in toxicity evaluation and the limited physicochemical data reported for most of the nanomaterials makes it hard to determine their safe use [7,8]. Specifically, silver nanoparticles (AgNPs) are among the most studied and applied nanomaterials in the biomedical field [9]. The benefit and toxic effects of silver nanoparticles have been related to their physicochemical properties such as their size, shape, coating, and zeta potential [10]. One of the main suggested contributors for biological effects elicited by AgNP is Ag^+^ ions release, which induces reactive oxygen species (ROS), leading to a redox imbalance [11]; nevertheless, the full mechanisms of action for AgNPs still unclarified. 

Recently, many research groups, including ours, determine that the entire nanoparticle and not the silver ion release were the foremost responsible for the ROS overproduction that leads the cells exposed to AgNPs to death [12,13,14,15]. ROS overproduction is an effective strategy employed to eliminate or decrease high-proliferate culture growth sensitive to redox imbalance such as parasites and tumors [16,17,18,19]. In Mexico, both high-proliferate diseases are public health problems. Tumors are the fourth cause of death; meanwhile, parasitic diseases affect children under one year of age and people with compromised immune systems, according to National Institute of Statistics and Geography (INEGI) data [20]. In the search for new alternatives for treatment, our research group has worked with a fully characterized PVP-AgNPs formulation that exhibits in vitro [21] and in vivo [22] antimicrobial activity, virucide effect [23,24,25], antiproliferative and antitumor activity [26,27], antiparasitic potential [28,29,30], and immunomodulatory capacity [31,32], among others during the last years. We found that Ag^+^ ions release seems not to be the primary mechanism of action. It was described that the [coating agent]/[metal] ratio could be one the most relevant parameter that confers Argovit^®^ formulation specificity against pathogens and malignant cells with neither cytotoxic nor genotoxic damage on healthy cells [14,15].

In order to improve the biocompatibility of AgNPs formulation and determine if the [coating agent]/[metal] ratio plays a significant role in the selectivity of AgNPs biological activity, we evaluate the antiproliferative activity of a new protein-coated-AgNPs formulation on cell cultures of human colorectal adenocarcinoma cells (HCT-15), *Entamoeba histolytica* HM1-IMSS trophozoites, and mice primary cultures, i.e., liver, spleen, kidney, and brain BALB/c. We determined the genotoxic potential of this formulation through micronuclei frequency on human peripheral blood lymphocytes (HPBL) using the cytokinesis-block micronucleus (CBMN) assay, determining its median lethal dose (LD_50_) in BALB/c mice and performing a histopathological analysis. Our results showed that BioArgovit has a selective antiproliferative and antiparasitic activity without evidence of cytotoxic, genotoxic, or toxic adverse effects in vitro and in vivo for healthy systems. These findings open new opportunities in the formulation of selective and safe nanomaterial developed for biomedical applications.

## 2. Materials and Methods

### 2.1. Silver Nanoparticles (AgNPs) Characterization

Silver nanoparticles used in this work were donated by Dr. Vasily Burmistrov from the Scientific and Production Center Vector-Vita (Russia). BioArgovit^®^ is a hydrolyzed protein-coated AgNPs synthesized as described in the patent RU2646105C1 “Method for silver proteinate production.” This formulation is a highly dispersed water suspension with an overall 200 mg/mL (20%) concentration. The metallic silver content reported for this formulation is 111.2 mM (12 mg/mL) stabilized with 188 mg/mL of hydrolyzed protein.

Conventional spectroscopic techniques were used to characterize the batch used in this work. The hydrodynamic diameter and the zeta potential were determined using dynamic light scattering (DLS) (Malvern Instruments Zetasizer Nano NS model DTS 1060, λ = 532 nm). The optical properties were determined with a Cary 60 UV-Vis spectrophotometer (Agilent Technologies) range of 200 to 900 nm. Morphology and size distribution were determined by HR-TEM using a JEOL-JEM-2010 microscope. A thermal analysis system SDT Q600 was employed to perform the thermogravimetric analysis (TGA) and the differential scanning calorimetry (DSC) under argon atmosphere from 30 to 750 °C at a rate of 10 °C min^−1^.

All dilutions of AgNPs prepared in this work were performed with distilled and sterile water. Silver content obtained from TGA analysis was considered to get the working solutions. Solutions prepared were kept at 4 °C in darkness for no more than 72 h; after that time, new dilutions were prepared. From here on, all concentrations named in this work correspond to the amount of metallic silver contained in the AgNPs formulation.

### 2.2. In Vitro Antitumor Activity 

Human colorectal adenocarcinoma cells HCT-15 (Duke’s type C) were acquired from American Type Culture Collection (ATCC^®^ CCL-225™). The cells were kept until 80% confluence in RPMI-1640 with l-Glutamine media (Biowest L0500) supplemented with 10% (*v/v*) of heat-inactivated Fetal Bovine Serum (FBS, Biowest S1650, Nuaillé, France) and 0.01% antibiotic–antimycotic (Streptomycin and penicillin G, Biowest L0010) at 37 °C in a humidified atmosphere with 5% CO_2_. Sub-culturing was performed every day. Cells from the third sub-culture and beyond were used to determine the antiproliferative effect of AgNPs.

HCT-15 cells were seed on a 96-well culture plate; each well contains 1 × 10^4^ cells, 190 µL of fresh supplemented RPMI-1640 media, and 10 µL of the different AgNPs concentrations, namely 5.56 µM, 55.6 µM, 556.2, µM and 5.56 mM of metallic silver (0.6, 6, 60, and 600 µg/mL of metallic silver). HCT-15 cells were exposed to AgNPs for 24 h; the nanoparticles were added just after the cells were placed in the well. Cell viability was determined by trypan blue vital stain with an automatic cell counter and then confirmed by flow cytometry. Once the half-inhibitory concentration (IC_50_) was determined, this concentration, a half, and double of it were used to evaluate the capacity of AgNPs to increase the concentration of reactive oxygen species (ROS) within the cell and on mitochondria and to identify the cell death pathway induced (apoptosis or necrosis). Carboplatin (190 µM; 70.53 µg/mL) [33] was used as a positive control, while HCT-15 cells without treatment were employed as the negative control. 

ROS quantification within the cells was performed with DCFDA/H_2_DCFDA—Cellular ROS Assay Kit (Abcam ab113851, Cambridge, MA, USA). MitoSOX™ Red mitochondrial superoxide indicator for a live-cell imaging kit (Invitrogen M36008, Carlsbad, CA, USA) was used to quantify superoxide radical anion concentration within mitochondria. Dead Cell Apoptosis Kit with Annexin V Alexa Fluor 488™ and Propidium Iodide (Invitrogen V13241, Waltham, MA, USA) was used to identify apoptosis/necrosis processes. All determinations were done following the Kits provider instructions on an Attune NxT flow cytometry equipment. 

### 2.3. In Vitro Amoebicidal Activity 

*Entamoeba histolytica* trophozoites (HM1:IMSS) were axenically grown in TYI-S33 medium supplemented with 10% (*v*/*v*) of heat-inactivated Adult Bovine Serum (ABS), 5% (*v*/*v*) of Diamond vitamins, and 1% (*v*/*v*) of antibiotic until 80% confluence; then, they were sub-cultured twice prior to their use for antiproliferative assays. Trophozoites were seeded at 37 °C and 5% CO_2_ with a density of 6 × 10^3^ trophozoites per well in a 96-well culture plate and exposed for 24 h to different concentrations of AgNPs. The AgNPs were added just after the trophozoites placed in the well. Lack of cell adherence is considered a cytotoxic effect. The final concentration of AgNPs used were: 27.8, 55.6, and 111.2 µM considering metallic silver content (3, 6, and 12 µg/mL). Untreated parasites and metronidazole (Mtz = 7 µM; 0.82 μg/mL) were the negative and positive control, respectively. Viability was determined by the trypan blue exclusion test using a Neubauer chamber under an optical microscope after 72 h of incubation at 37 °C and 5% CO_2_ in anaerobic conditions. Three independent experiments were performed by triplicate.

### 2.4. Toxicity Evaluation

#### 2.4.1. In Vitro Studies

##### Primary Cell Culture Isolation and Viability

Primary cultures of the liver, spleen, kidney, and brain were isolated from healthy BALB/c mice. Briefly, obtained organs were perfused with physiological saline solution and subsequently washed with the same solution. Primary cell cultures were seeded on Petri dishes with RPMI 1640 media supplemented with 10% (*v*/*v*) of heat-inactivated Fetal Bovine Serum (FBS) and incubated at 37 °C with 70% of relative humidity and 5% CO_2_.

The cytotoxic effect of AgNPs on primary cell cultures of healthy BALB/c mice was performed by exposing the isolated cells for 24 h to different concentrations of this AgNPs formulation. Then, 5 × 10^4^ cells seeded in a 96-well culture plate were exposed to AgNPs at final concentrations of 5.5 µM, 55.6 µM, 556.2 µM, and 5.5 mM of metallic silver (0.6, 6, 60, and 600 µg/mL); untreated cells were used as a negative control. All cultures were incubated with the corresponding stimulus at 37 °C and 5% CO_2_ for 24 h. Cells were counted with an optical microscope using a Neubauer chamber and the vital exclusion dye trypan blue. Three independent experiments by triplicate were performed.

##### Micronuclei Frequency on Human Peripheral Blood Lymphocytes

Cytokinesis-block micronucleus assay (CBMN) was used as described by Fenech [34] to quantify the micronuclei frequency in lymphocytes from a healthy donor (male, 38 years). The final concentrations of AgNPs used in this assay were 0.1, 1.1, 11, and 111 µM of metallic silver (0.012, 0.12, 1.2, and 12 μg/mL) contained on the AgNPs formulation. Briefly, 0.5 mL of heparinized peripheral blood was mixed with 6.3 mL of HEPES RPMI-1640 modified medium (Sigma R5886, St. Louis, MO, USA). The latter was previously supplemented with 1% L-glutamine (Sigma G6392, St. Louis, MO, USA), 1% of non-essential amino acids (Sigma M7145, St. Louis, MO, USA), and 0.2 mL of phytohemagglutinin 1 mg/mL (Sigma L2646, St. Louis, MO, USA). After 44 h of incubation at 37 °C, 21 μL of 2 mg/mL cytochalasin B (Sigma C6762, St. Louis, MO; USA) was added to the mixture to blocks cell cytokinesis. The cultures were incubated for another 28 h and then harvested through centrifugation, fixed with methanol (Sigma-Aldrich 34860, St. Louis, MO; USA)-acetic acid (Sigma-Aldrich ARK2183, St. Louis, MO; USA) solution, and placed on a slide for later staining with eosin b (Sigma 2853, St. Louis, MO; USA) and methylene blue (Sigma O3978, St. Louis, MO; USA). Cells were counted in a light field microscope (Primo Star Zeiss) with a 100× objective lens. 

The cytokinesis-block proliferation index (CBPI) and replication index (RI) were calculated counting 1000 cells, including mononuclear (MONON), binucleated (BN), and trinucleated and tetranucleated (MULTIN) cells according to (1) and (2), respectively:

(1) $$CBPI=\;\frac{\left(\#\;MONON\;cells\right)+\left(2*\#\;BN\;cells\right)+\;\left(3*\#\;MULTIN\;cells\right)}{Total\;number\;of\;cells},$$

(2) $$RI=\;\frac{{\left[\left(\#\;BN\;cells\right)+\left(2*\#\;MULTIN\;cells\right)\right]}_{T\;}/{\left(Total\;\#\;of\;cells\right)}_{T}}{{\left[\left(\#\;BN\;cells\right)+\left(2*\#\;MULTIN\;cells\right)\right]}_{C\;}/{\left(Total\;\#\;of\;cells\right)}_{C}},$$

where *T* = test chemical treatment culture and *C* = control culture.

The cytostasis percentage is defined in (3) as follows:

(3) $$\%\;Cytostasis=\;100-100\;\times \;\left(\frac{{\left[CBPI\right]}_{T}\;-\;1}{{\left[CBPI\right]}_{C}\;-\;1}\right).$$

The nuclear division index was determined for comparative purposes according to (4):

(4) $$NDI=\;\frac{\left(\#\;MONON\;cells\right)\;+\;\left(2*\#\;BN\;cells\right)\;+\;\left(3*\#\;TRIN\;cells\right)\;+\;\left(4*\#\;TETRAN\;cells\right)}{Total\;\#\;of\;cells}.$$

The genotoxic effect of each treatment was evaluated by determining micronuclei frequency (MN), nuclear outbreaks (NBUDs), and chromatin bridges (NPBs) on 1000 binucleated cells (*BN*) with well-preserved cytoplasm. The same number of cells were used to quantify apoptosis and necrosis.

### 2.5. In Vivo Studies 

#### 2.5.1. Lethal Dose

The lethal dose of AgNPs was determined on BALB/c mice following the OECD Guideline 420 for Acute Oral Toxicity Assay [35]. The Ethical Committee of the Autonomous University of Baja California approved the experimental protocol (001/2018), Mexico. A sighting study was carried out to select the appropriate starting dose. One BALB/c female mouse was administered with 0.4 mL of the concentrate solution of AgNPs (44.5 µmol (4.8 mg)) of metallic silver) and kept in observation for 14 days. Changes in mice clinical signs such as skin and fur appearance, eyes, membranes, conduct, respiratory pattern, the presence of nausea, vomiting, diarrhea, tremors, convulsions, salivation, lethargy, sleep, coma, and death were carefully registered. Since no sign of toxicity was observed after the administration of 0.4 mL of AgNPs, the limit test with six female BALB/c mice was performed. Mice of eight weeks old and an average weight of 22 g were assigned to polycarbonate cages maintained at 25 °C, 60% humidity, 12/12-h light–dark cycle. Mice were administered with 0.4 mL AgNPs every two hours for a total of 12 administration in a period of 24 h. The final amount of metallic silver administered after 24 h was 534 µmol (57.6 mg), corresponding to 24.2 mmol (2618 mg) of metallic silver/Kg of body weight. The administered mice were kept in an observational period of 14 days. Food and water were available ad libitum. Additionally, all animal glucose levels were monitored, taking a blood drop from the tail during the limit test experiment. 

After completing the observational period, Ketamine (100 mg/Kg body weight, Ketamin-Pet, Aranda Laboratories, Querétaro, MEX) and Xylazine (Procin Equus, Pisa, Hidalgo, MEX) were used to anesthetize mice, which were posteriorly exsanguinated by cardiac puncture. Blood was collected and centrifuged (Heraeus Fresco 21, Thermo Scientific, Osterode, Germany) at 2500 rpm for 10 min to separate the serum. The liver, kidney, spleen, lung, intestine, heart, and brain were removed and placed in 10% formaldehyde for histopathological evaluation. 

#### 2.5.2. Pathology

The histopathologic evaluation was performed as recommended by the OECD Guideline 420 [35]. Tissues were processed using routine histological techniques. Then, 3–5 µm sections were cut and stained with hematoxylin and eosin (H&E) for histopathologic evaluation. Tissue injury was examined microscopically for evidence of cellular damage and inflammation.

### 2.6. Statistics

Viability, apoptosis, necrosis, and ROS generation were analyzed with a two-way ANOVA (*p* < 0.05) followed by a Tuckey post hoc test (*p* < 0.05). Micronuclei and other nuclear anomalies fit well with the Poisson model. Therefore, to guarantee the homoscedasticity between the experimental groups, a square root transformation was carried out according to formulae:

(5) $$\hat{X}=\;\sqrt{\left(X+0.5\right)},$$

where $\hat{X}=$ estimator of the transformed variable, *X* = original variable, number of counted anomalies. A Kruskal–Wallis test was performed with Tukey’s post hoc multiple comparison test. All data were analyzed using GraphPad Prism 8 (GraphPad Software, San Diego, CA, USA). 

## 3. Results

### 3.1. Characterization of Silver Nanoparticles

**Figure 1:**
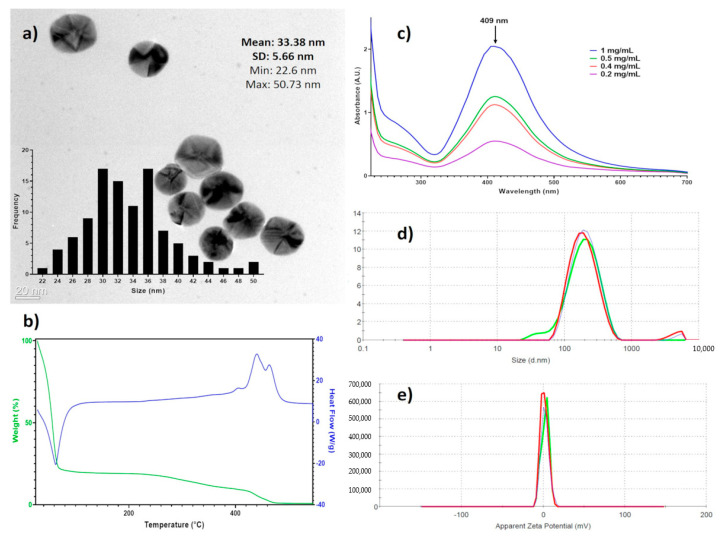
Physicochemical characterization of BioArgovit silver nanoparticles. (**a**) HR-TEM images that show the morphology of silver nanoparticles (The insert corresponds to the size distribution histogram); (**b**) the thermogravimetric analysis of the formulation (TGA) / differential scanning calorimetry (DCS); (**c**) surface plasmon resonance by UV–Vis; (**d**) hydrodynamic diameter determined by dynamic light scattering (DLS), and (**e**) the zeta potential.

Spectroscopic techniques were used to characterize entirely the AgNPs batch used in this work. Figure 1a shows that the AgNPs are mainly spherical, with a size average of 33.3 ± 5.6 nm as determined by HR-TEM. The DSC-TGA results (Figure 1b) show 80.4% mass loss at 116 °C corresponding to water in the formulation, at 423 °C begins the decomposition of the coating agents finishing at 470 °C (18.1%), and the remaining mass corresponds to metallic silver (1.4%). These results agree with data of manufacturer, 18.8% of the coating agent and 1.2% of metallic silver. The UV-vis analysis showed the plasmon surface resonance at 409 nm (Figure 1c). The hydrodynamic diameter, summarizing the diameter of the metallic silver nanoparticle and hydrolyzed protein coating, is determined by DLS is 165.5 ± 105 nm (Figure 1d) and the ζ potential of 2.3 ± 4.7 mV (Figure 1e).

### 3.2. In Vitro Antitumor Activity on HCT-15

**Figure 2:**
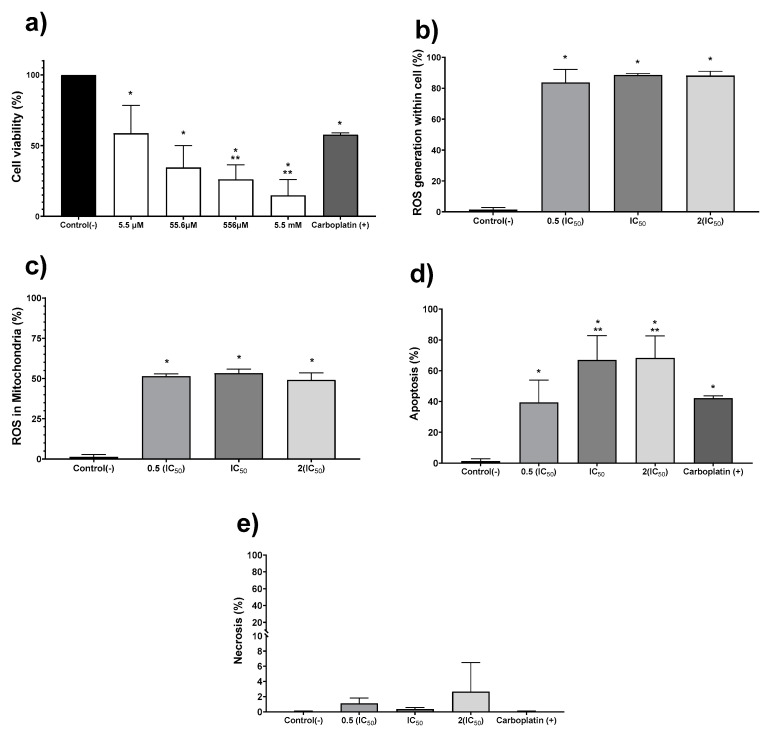
Silver nanoparticles (AgNPs) antiproliferative activity on human adenocarcinoma HCT-15. Cell viability (**a**) was determined using 5.5 µM, 55.6 µM, 556.2 µM, and 5.5 mM (0.6, 6, 60 and 600 μg/mL) of metallic silver contained in AgNPs. (**b**) Apoptosis, (**c**) necrosis, (**d**) reactive oxygen species (ROS) overproduction within the cell, and (**e**) ROS overproduction inside mitochondria were determined using half of the IC_50_, IC_50_, and double of the IC_50_. The * represents a significant difference (*p* ≤ 0.001) compared with negative control, ** represents a significant difference (*p* ≤ 0.001) compared with carboplatin.

The in vitro antiproliferative activity on human colon tumor cell line HCT-15 was evaluated exposing the cell culture to the AgNPs formulation for 24 h. The concentration range assessed was 5.5 µM to 5.5 mM (0.6 to 600 μg/mL), obtaining a half-inhibitory concentration (IC_50_) of 19.7 µM (2.1 μg/mL) (Figure 2a). Once the IC_50_ for AgNPs was determined, further experiments to quantify ROS overproduction, apoptosis, and necrosis were performed with the IC_50_, one-half, and double this concentration (Figure 2b–e). Regardless of the AgNPs concentration used, the ROS overproduction is close to 80% of positive events in all cases (Figure 2b), showing a significant statistical difference (*p* ≤ 0.001) compared with the negative control. Almost two-thirds of the quantified ROS are superoxide radical anion within the mitochondria, as shown in Figure 2c. It seems that ROS overproduction within mitochondria (Figure 2c) should trigger the intrinsic apoptosis pathway, generating 40% positive apoptosis events with half of the IC_50_ and close to 70% when the IC_50_ or double of this concentration were administered to tumor cells (Figure 2d). Half of the IC_50_ determined for AgNPs induces practically the same number of apoptotic events (39.3%) as the first-choice chemotherapeutic drug carboplatin (42.1%). In comparison, AgNPs IC_50_ or double this amount produces a 25% increase on the apoptotic events (Figure 2d). No evidence of necrosis was observed for any of the concentrations assessed (Figure 2e). In our experimental conditions, the IC_50_ value reported for carboplatin (190 µM; 70.5 µg/mL) [33] produces a cell viability decrease of 42.2%. In addition, this compound produces 42% of apoptosis events without necrosis evidence. 

### 3.3. Amoebicidal Activity

**Figure 3:**
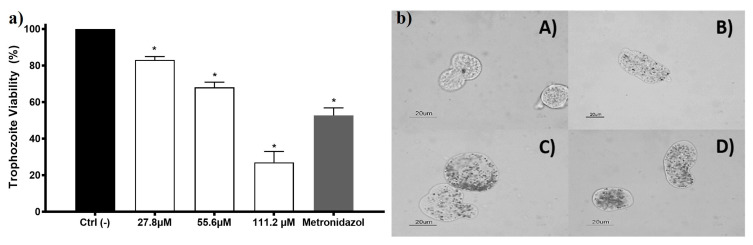
The amoebicidal activity of AgNPs against trophozoites of *Entamoeba histolytica*. *E. histolytica* trophozoite’s viability exposed to different concentrations of AgNPs 27.8, 55.6, and 111.2 µM of metallic silver (3, 6, and 12 µg/mL) and metronidazole (7 µM; 0.82 µg/mL) is shown in the left panel (**a**). The right panel (**b**) shows the micrographs of trophozoites (**A**) untreated and exposed to (**B**) 27.8 µM (3 µg/mL); (**C**) 55.6 µM (6 µg/mL), and (**D**) 111.2 µM (12 µg/mL) of metallic silver contained in AgNPs. The * represents a significant difference (*p* ≤ 0.05) compared with the negative control.

*Entamoeba histolytica* trophozoites viability exposed to AgNPs shows concentration-dependent behavior. Since the lowest concentration was assessed, a growth inhibition effect was observed (viability of 83%). The half-inhibitory concentration (IC_50_) for this AgNPs formulation on *E. histolytica* is 69.2 µM (7.4 µg/mL). The internalization of AgNPs in the trophozoite could be observed using a bright field microscope, and it appears to exhibit concentration-dependent behavior (Figure 3, left panel). Some of the nanoparticles were unequivocally seen within the trophozoite cytoplasm, confirming the internalization when the nanoparticles moved as the trophozoite did. Trophozoites exposed to AgNPs increase their size and membranes become thin, apparently related to the AgNPs increased concentration (Figure 3). These morphological changes are also observed in trophozoites exposed to compounds that elicit ROS overproduction [19,36,37].

### 3.4. Cell Viability of Mice Primary Cultures Exposed to AgNP

Several organs (brain, liver, kidney, and spleen) have been identified as target organs after administering different AgNPs formulations [38,39]. Therefore, we evaluate the viability of mice primary cultures of the organs mentioned above exposed to different concentrations of the AgNPs formulation studied in this work. The concentration range used in this essay, 5.5 µM to 5.5 mM (0.6 to 600 μg/mL) of metallic silver, includes the IC_50_ values found on the in vitro antitumor and amoebicidal activity trials. 

**Figure 4:**
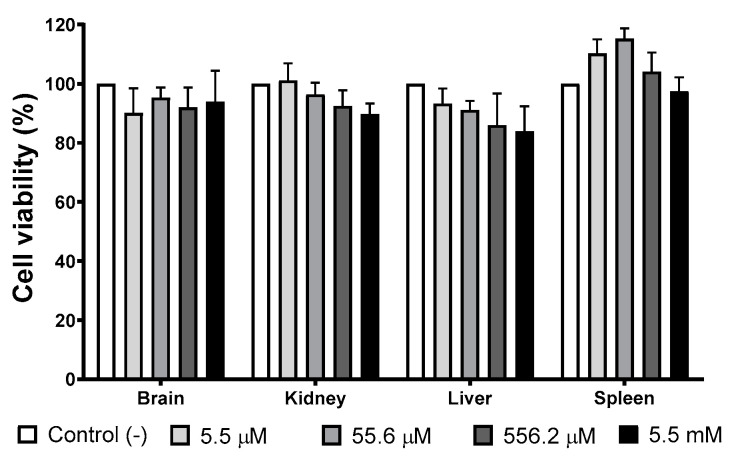
AgNPs’ effect on selected primary cultures cell viability. Brain, kidney, liver, and spleen primary cultures obtained from BALB/c mice were exposed to 5.5 µM, 55.6 µM, 556.2 µM, and 5.5 mM (0.6, 6, 60, and 600 μg/mL) for 24 h. For each cell type, the primary culture without treatment was used as a negative control.

Figure 4 shows the cellular viability of primary cultures exposed for 24 h to different concentrations of AgNPs. There is no evidence of growth inhibition effect elicited by AgNPs exposure at any of the concentrations assessed compared with the negative control. Low concentrations of AgNPs (5.5 and 55.6 µM of Ag; 0.6 and 6 μg/mL of Ag) promote spleen cultures’ growth, but no statistically significant differences were found with the negative control. 

### 3.5. Cytokinesis-Block Micronucleus Assay (CBMN)

To complete the in vitro cytotoxic behavior of this AgNPs formulation, we perform the cytokinesis-block micronucleus (CBMN) assay with human peripheral blood lymphocytes (HPBL). This assay not only allows us to determine the potential genotoxic damage but also record cytotoxic biomarkers such as the proliferation capacity of the cells, apoptosis, and necrosis. HPBL were exposed to the same concentration range as that used on mice primary cultures, 0.1 to 111 µM (0.012 to 12 µg/mL) of metallic silver.

**Figure 5:**
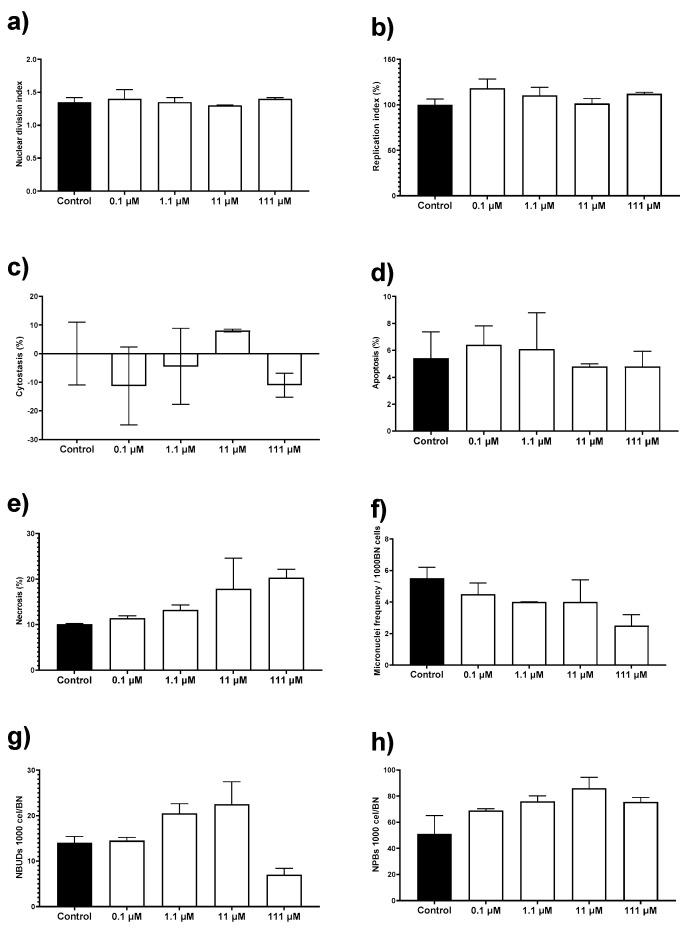
Evaluation of cytotoxic and genotoxic effects of different concentrations of AgNPs on human peripheral blood lymphocytes (HPBL). HPBL were exposed to 0.1, 1.1, 11, and 111 µM (0.012, 0.12, 1.2, and 12 µg/mL) following the cytokinesis-block micronucleus (CBMN) assay registering the biomarkers (**a**) nuclear division index; (**b**) replication index; (**c**) cytostasis; (**d**) apoptosis; (**e**) necrosis; (**f**) micronuclei frequency; (**g**) nuclear buds (NBUDs); and (**h**) nucleoplasmic bridges (NPBs).

The AgNPs formulation used in this work results neither cytotoxic nor genotoxic for HPBL at the concentration range assessed. No changes were observed on the nuclear division index (NDI), replication index (RI), cytostasis, and apoptosis by exposure to AgNPs compared with the negative control (Figure 5a–d). There is an increase of 10% in necrosis compared with the control, but the difference was not statistically significant (Figure 5e). None of the concentrations assessed generates a micronuclei frequency higher than the observed for the negative control (Figure 5f). The frequency of nuclear buds (NBUDs) and nucleoplasmic bridges (NPBs) is almost double on lymphocytes exposed to 11 and 111 µM (1.2 and 12 µg/mL) of metallic silver contained in AgNPs (Figure 5g–h) when compared with the negative control. Despite that, a Kruskal–Wallis analysis did not show statistically significant differences between the control group and the AgNPs concentrations assessed.

It is essential to mention that the AgNPs concentration range used to evaluate the cytotoxic and genotoxic damage on lymphocytes include the IC_50_ values found in the antiproliferative activity assays against human tumor cell lines (19.7 µM; 2.1 µg/mL) and *E. histolytica* trophozoites (69.2 µM; 7.4 µg/mL). Therefore, concentrations that effectively inhibit the proliferation of human colon tumor cells and *E. histolytica* trophozoites are neither cytotoxic nor genotoxic for HBPL in vitro. 

### 3.6. In Vivo Studies

#### 3.6.1. Determination of Lethal Dose

The lethal dose of AgNPs determined on BALB/c mice according to the Guideline 420 for Acute Oral Toxicity Assay from the OECD shows the low toxicity of this formulation, since all mice that were administered with 24.2 mmol of metallic silver (2618 mg of metallic silver) per Kg of body weight in a period of 24 h survive the observational period of 14 days. The aforementioned dose, 2618 mg of metallic silver/Kg, places this formulation in Category 5 according to the Globally Harmonized Classification (GHS) [40]. Only one mouse of the five show signs of lethargy and hunching after the third administration of 0.4 mL of AgNPs, which is equivalent to a cumulative amount of 133.5 µmol (14.4 mg) of metallic silver on the mice. The mouse also presented bilateral eye discharge after the fifth administration. However, the mouse survived the administration and 24 h later presented no signs of toxicity. Diarrhea was observed in all mice after administration of 222.5 µmol (24 mg) of metallic silver. Ruffed hair and dehydration were observed after 400.5 µmol (43.2 mg) of metallic silver. These conditions remained up to the end of the limit test, that is, the administration of 534 µmol (57.6 mg) of metallic silver per mice (24 h). Nevertheless, as mentioned before, all mice survived the administration process and the 14-day observational period. All mice ate and drank normally 24 h after the end of the administration of AgNPs.

At the end of the observational period, glucose (GLU), alkaline phosphatase (ALP), aspartate aminotransferase (AST), and alanine aminotransferase (ALT) levels were quantified on blood collected by heart puncture. Table 1 compiles the four biomarkers values found on mice administered with AgNPs. Except for AST, which presents a significant increase in its concentration, the other biomarkers were within the reported range for BALB/c mice compared with the control group.

#### 3.6.2. Pathology Results 

**Figure 6:**
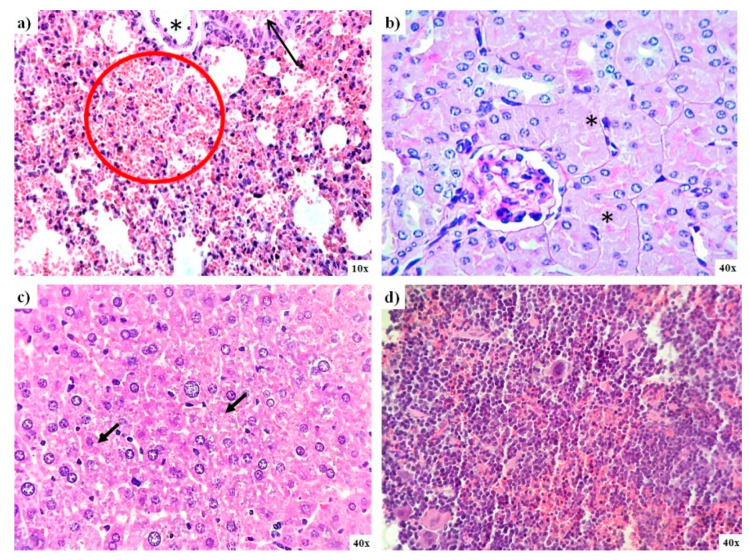
Lung, kidney, liver, and spleen pathology analysis of mice exposed to AgNPs from lethal dose determination. (**a**) Small bleeding patches (red circle) and bronchiolar lumen (black arrow) found in the lung; (**b**) necrosis found in kidney tubules (asterisk); (**c**) evidence of microvesicular steatosis in the liver (black arrows), and (**d**) spleen tissue that exhibits light signs of congestion.

Histological analysis of organs from mice exposed to AgNPs during the limit test confirms the low toxicity exerted by AgNPs. In general, no significant differences were found between mice from the control group and those administered with AgNPs. Nevertheless, diffuse damage observed on the different tissues analyzed were compiled in Table 2. Small bleeding patches were observed on the lungs, and there were signs of focal acute tubular necrosis on kidney and liver steatosis (Figure 6a–c). The spleen of these animals also exhibits diffuse signs of congestion, and two-thirds of mice display hematopoiesis; however, the latter condition is typical in this species (Figure 6d). Two-thirds of this animal brain exhibit signs of ischemia; only one-third shows capillary congestion on the intestine, and the heart tissue analyzed present no damage (Table 2). 

## 4. Discussion

The versatility of AgNPs in the biomedical field has been demonstrated and discussed in several recent reviews [6,41,42,43]. Their antimicrobial, antiviral, antiparasitic, and antiproliferative activity relies on physicochemical properties such as size, surface charge, and coating agent [39,44,45,46,47]. The use of PVP as an AgNPs coating agent reduces the cytotoxic and genotoxic effects observed in other formulations with similar size but uncoated or stabilized with citrate [12,48,49,50,51]. One of the mechanisms suggested for the biological response elicited by AgNPs is ROS overproduction [27,44,47,52,53,54], which is associated with silver ion release from unstable formulations or those produced by the entire nanoparticle [12,14]. ROS overproduction can be used to eliminate or reduce susceptible growth to redox imbalances cultures such as tumors and parasites [16,55,56].

In this work, a new protein-coated AgNPs formulation named BioArgovit^®^ has been fully characterized. The AgNPs studied here present a spherical shape with an average diameter of metallic silver particle of 33.38 ± 5.66 nm (measured with HR-TEM) and silver particle with the protein coat and solvation sphere of 165.5 ± 105 nm determined with DLS. The silver and hydrolyzed protein content is 1.4% and 18.1% of the total formulation weight, respectively. The plasmon resonance was found at 409 nm, with a zeta potential of 2.3 ± 4.7 mV. The physicochemical properties of this new formulation are quite similar to those found for a PVP-AgNPs formulation previously studied by our research group [14,15,27,31,57,58,59].

A direct relationship between the human adenocarcinoma cell viability and ROS overproduction was observed. ROS have mainly overproduced in the mitochondria of HCT-15 cells. The mitochondrial ROS overproduction could be responsible for triggering the intrinsic cell death pathway [60] that finally leads to the high amount of apoptosis events registered on HCT-15 cells (Figure 2d). The behavior mentioned above was already described on human tumor cervix cells (HeLa and SiHa) exposed to *GinKgo-biloba*-AgNPs [61] and on murine melanoma (B16F10) [27] and human tumor cells of the cervix (HeLa) and breast (MCF-7 and MDA-MB-231) exposed to different concentrations of PVP-AgNPs [26].

The AgNPs formulation studied here has an IC_50_ = 19.7 µM (2.1 µg/mL) against HCT-15 cells, which means an antiproliferative potency almost 10 times higher compared with carboplatin (190 µM; 70.5 µg/mL), a first-line chemotherapeutic agent. Carboplatin lacks nephrotoxicity and shows less emetic activity than cisplatin, but 5 µg/mL of this compound shows a significative cytotoxicity, increase of sister chromatid exchange (SCE), and MN frequency increase in human lymphocytes [62], which are effects that eventually lead to myelosuppression [63,64]. As shown by our results, neither of both BioArgovit^®^ nor Carboplatin elicit necrosis but induce cell death mainly by apoptosis (Figure 2d,e). In contrast to Carboplatin, BioArgovit^®^ do not produces neither cytotoxic nor genotoxic damage on peripheral blood human lymphocytes, even with more than twice the concentration required of carboplatin to produce genetic material damage (Figure 5a–e). These results suggest the low genotoxic potential of this AgNPs formulation and agree with the lack of cytotoxicity found in mice primary cultures of different organs (Figure 4) as well as the very low damage evidenced by histopathology in several tissues of mice exposed to a high amount of AgNPs such 2618 mg of Ag/Kg of body weight (24.2 mmol Ag^0^/Kg of body weight), as shown in Figure 6.

ROS overproduction could be one of the main pathways that lead to the death of *E. histolytica* trophozoites exposed to protein-coated AgNPs formulation. ROS overproduction was already reported as a death promoter of marine parasites exposed to PVP-AgNPs [29,30]. The anti-helminthic effect of garlic clove extract-AgNPs [65] and the antimalarial activity of *Dicoma Anomala* Sond. root extract-AgNPs [66] were also associated with ROS overproduction. It is the first time that amoebicidal activity against *Entamoeba histolytica* trophozoites has been reported to our knowledge.

The AgNPs formulation evaluated herein shows selective cytotoxicity. No cytotoxic damage was observed in murine primary cultures (brain, kidney, liver, spleen) with the concentration range assessed (5.5µM to 5.5 mM; 0.6 to 600 µg/mL). Neither genotoxic nor genotoxic damage was observed on HPBL exposed to the same concentrations range. There is no difference in micronuclei, nucleoplasm bridges, or nuclear budge compared with negative control according to the CBMN assay. The AgNPs concentration of 111 µM (12 µg/mL) is almost twice and more than 5 times higher than the concentration needed to inhibit *E. histolytica* trophozoites (69.2 µM; 7.47 µg/mL) and human adenocarcinoma (19.7 µM; 2.13 µg/mL) proliferation, respectively.

There is no change in mice primary cell viability compared with the negative control, just a small increase non-statistically different on spleen cultures exposed to 5.5 and 55 µM of AgNPs (0.6 and 6 µg/mL). The mentioned increase could be related to the immunological response elicited by AgNPs leading to lymphocyte proliferation. The result agrees with immunological stimulation reported for different AgNPs formulations on diverse organisms [32,67,68,69,70,71]. The selective toxicity of AgNPs formulation studied in this work was also confirmed with the histopathological analysis and the lethal dose. After the administration of AgNPs 2618 mg Ag/Kg of body weight (24.2 mmol Ag^0^/Kg of body weight), no dead or abnormal behavior during or after the observational period of 14 days was observed, only diffuse damage on selected organs, namely the kidney, liver, lung, spleen, brain, intestine, and heart (Table 2 and Figure 5). Levels within the average range of biochemical biomarkers such as ALT, AST, APT, and glucose also suggest high biocompatibility (Table 1). Elevated AST levels (Table 1) without evidence of damage to tissues such as the liver, spleen, intestine, and kidney (Figure 6) are associated with experimental animal manipulation instead of the evaluated agent toxicity [72].

Likewise, Kim [73] and Maneewattanapinyo [74] reported AgNPs formulations with lethal doses determined on murine models of 2000 and 5000 mg/Kg of body weight, respectively. These formulations also can be classified on GHS Category 5 (practically nontoxic). Unfortunately, scarce physicochemical information was reported to compare with the formulation studied in this work directly. On the other hand, the protein-coated AgNPs formulation’s physicochemical properties studied here are similar to previously studied PVP-AgNPs formulation properties. However, doses evaluated to determine the toxic response of protein-coated AgNPs are three orders of magnitude higher than the used for PVP-AgNPs, from 2.2 to 2618 mg of metallic silver/Kg of body weight [75], strongly suggesting the biocompatibility improvement obtained by the change of coating agent. Therefore, size, shape, and [coating agent]/[metal] ratio are crucial elements for designing effective and safe AgNPs.

## 5. Conclusions

This work reports a new protein-coated AgNPs formulation entirely characterized by spectroscopic techniques. AgNPs show a spheroidal shape with an average of 33.3 ± 5.6 nm, the metallic silver content of 1.4% (*w*/*w*), and 18.1% (*w*/*w*) of a protein coating. BioArgovit^®^ show an IC_50_ = 19.7 µM (2.1 µg/mL) on human adenocarcinoma HCT-15 cells, which represent almost 10 times more potency to inhibit the growth of these highly aggressive tumor cells than the first-line chemotherapeutic agent, carboplatin (190 µM, 70.5 µg/mL). The main cell death pathway induced is apoptosis, which is probably triggered by ROS overproduction quantified mainly on mitochondria. No evidence of necrosis was observed. In addition, neither cytotoxic nor genotoxic damage was observed on human peripheral blood lymphocytes exposed to 111 µM (12 µg/mL) of this AgNPs formulation, which is almost 10 times the concentration of carboplatin (13.4 µM; 5 µg/mL) that produces genotoxic damage. The foregoing suggests that BioArgovit^®^ improves the antiproliferative potency on HCT-15 cultures and cytotoxic selectivity both ten times more than carboplatin.

This AgNPs formulation also presents moderate antiproliferative activity against HM1-IMSS *Entamoeba histolytica* trophozoites cultures, with an IC_50_ = 69.2 µM (7.4 µg/mL). The antiproliferative mechanism seems also to be associated with ROS overproduction.

The low toxicity of this formulation was confirmed in different models. No change in cell viability on mice primary cultures of brain, liver, spleen, and kidney exposed to AgNPs concentration range from 5.5 µM to 5.5 mM (0.6 to 600 µg/mL of Ag) was observed. At the same concentration range of AgNPs, no cytotoxic or genotoxic damage was observed on human peripheral blood lymphocytes. The lethal dose was determined following the OECD guideline 420 for Acute Oral Toxicity Assay, obtaining an LD_50_ ≥ 2618 mg of Ag/Kg body weight (24.2 mmol Ag/Kg of body weight). All mice survived the observational period, the histopathology and biochemical analysis show no differences compared with the negative control group. Thus, all results from toxicological evaluation suggest a Category 5 (practically nontoxic) of the Globally Harmonized System of Classification and Labelling of Chemicals for this specific protein-coated AgNPs formulation after oral administration for a short period and urge the completion of its preclinical toxicological profile.

The results obtained in this work showed that BioArgovit^®^ AgNPs formulation has a selective antiproliferative and antiparasitic activity without evidence of cytotoxic, genotoxic, or toxic adverse effects in vitro and in vivo for healthy systems. These findings open new opportunities in the development of selective, safe, and effective AgNPs formulations for the treatment of cancer and parasitic diseases with a significant reduction of side effects.

## Figures and Tables

**Table 1:** Biochemical biomarkers levels of mice exposed to AgNPs during the limit test assay. Alkaline phosphatase (ALP), aspartate aminotransferase (AST), Alanine aminotransferase (ALT), and Glucose (Glu) concentrations were quantified and compared with reference values of BALB/c mice.

		Values from Limit Test			Reference Values			
Analyte	Units	Mean	SD ^a^	N ^b^	Mean	Low	High	N ^b^
ALP	U/L	305	229	5	187	108	367	138
ALT	U/L	127	193	5	63	40	170	121
AST	U/L	552	630	5	154	67	381	130
Glucose	mg/dL	136	18	5	193	85	281	132

^a^ SD = Standard Deviation; ^b^ N = number of animals.

**Table 2:** Histopathological analysis of kidney, liver, lung, spleen, brain, intestine, and heart of mice exposed to AgNPs.

Tissue	Pathological Finding	Incidence
Kidney	Tubular necrosis data	6/6
Liver	Micro vesicular steatosis and capillary congestion	6/6
Lung	Small bleeding patches	6/6
Spleen	Capillary congestion and hematopoiesis	6/6 and 4/6, respectively
Brain	Signs of ischemia	4/6
Intestine	Capillary congestion	2/6
Heart	Near normal	6/6

## Data Availability

Not applicable.

## References

[1] Pelgrift R.Y., Friedman A.J. (2013). Nanotechnology as a therapeutic tool to combat microbial resistance. Adv. Drug Deliv. Rev..

[2] Rai M., Deshmukh S.D., Ingle A.P., Gupta I.R., Galdiero M., Galdiero S. (2016). Metal nanoparticles: The protective nanoshield against virus infection. Crit. Rev. Microbiol..

[3] Awasthi R., Roseblade A., Hansbro P.M., Rathbone M.J., Dua K., Bebawy M. (2018). Nanoparticles in Cancer Treatment: Opportunities and Obstacles. Curr. Drug Targets.

[4] Rafique R., Kailasa S.K., Park T.J. (2019). Recent advances of upconversion nanoparticles in theranostics and bioimaging applications. TrAC-Trends Anal. Chem..

[5] Saji V.S., Choe H.C., Yeung K.W.K. (2010). Nanotechnology in biomedical applications: A review. Int. J. Nano Biomater..

[6] Ullah Khan S., Saleh T.A., Wahab A., Ullah Khan M.H., Khan D., Ullah Khan W., Rahim A., Kamal S., Ullah Khan F., Fahad S. (2018). Nanosilver: New ageless and versatile biomedical therapeutic scaffold. Int. J. Nanomed..

[7] Gomes A., Sengupta J., Datta P., Ghosh S., Gomes A. (2016). Physiological interactions of nanoparticles in energy metabolism, immune function and their biosafety: A review. J. Nanosci. Nanotechnol..

[8] Akhtar M.J., Ahamed M., Alhadlaq H.A. (2018). Challenges facing nanotoxicology and nanomedicine due to cellular diversity. Clin. Chim. Acta.

[9] Zhang X.-F., Liu Z.-G., Shen W., Gurunathan S. (2016). Silver Nanoparticles: Synthesis, Characterization, Properties, Applications, and Therapeutic Approaches. Int. J. Mol. Sci..

[10] Wei L., Lu J., Xu H., Patel A., Chen Z.S., Chen G. (2015). Silver nanoparticles: Synthesis, properties, and therapeutic applications. Drug Discov. Today.

[11] Xu L., Wang Y.Y., Huang J., Chen C.Y., Wang Z.X., Xie H. (2020). Silver nanoparticles: Synthesis, medical applications and biosafety. Theranostics.

[12] Ivask A., Voelcker N.H., Seabrook S.A., Hor M., Kirby J.K., Fenech M., Davis T.P., Ke P.C. (2015). DNA Melting and Genotoxicity Induced by Silver Nanoparticles and Graphene. Chem. Res. Toxicol..

[13] Cvjetko P., Milošić A., Domijan A.M., Vinković Vrček I., Tolić S., Peharec Štefanić P., Letofsky-Papst I., Tkalec M., Balen B. (2017). Toxicity of silver ions and differently coated silver nanoparticles in Allium cepa roots. Ecotoxicol. Environ. Saf..

[14] Ruiz-Ruiz B., Arellano-García M.E., Radilla-Chávez P., Salas-Vargas D.S., Toledano-Magaña Y., Casillas-Figueroa F., Luna Vazquez-Gomez R., Pestryakov A., García-Ramos J.C., Bogdanchikova N. (2020). Cytokinesis-Block Micronucleus Assay Using Human Lymphocytes as a Sensitive Tool for Cytotoxicity/Genotoxicity Evaluation of AgNPs. ACS Omega.

[15] Casillas-Figueroa F., Arellano-García M.E., Leyva-Aguilera C., Ruíz-Ruíz B., Vázquez-Gómez R.L., Radilla-Chávez P., Chávez-Santoscoy R.A., Pestryakov A., Toledano-Magaña Y., García-Ramos J.C. (2020). Argovit^TM^ silver nanoparticles effects on allium cepa: Plant growth promotion without cyto genotoxic damage. Nanomaterials.

[16] Nowak-Sliwinska P., Scapozza L., Altaba A.R.I. (2019). Drug repurposing in oncology: Compounds, pathways, phenotypes and computational approaches for colorectal cancer. Biochim. Biophys. Acta-Rev. Cancer.

[17] Poillet-Perez L., Despouy G., Delage-Mourroux R., Boyer-Guittaut M. (2015). Interplay between ROS and autophagy in cancer cells, from tumor initiation to cancer therapy. Redox Biol..

[18] Boros E., Dyson P.J., Gasser G. (2020). Classification of Metal-Based Drugs according to Their Mechanisms of Action. Chem.

[19] Toledano-Magaña Y., García-Ramos J.C., Torres-Gutiérrez C., Vázquez-Gasser C., Esquivel-Sánchez J.M., Flores-Alamo M., Ortiz-Frade L., Galindo-Murillo R., Nequiz M., Gudiño-Zayas M. (2017). Water-Soluble Ruthenium (II) Chiral Heteroleptic Complexes with Amoebicidal in Vitro and in Vivo Activity. J. Med. Chem..

[20] INEGI (2020). Comunicado De Prensa Núm. 480/20. (29/10/2020). Características De Las Defunciones Registradas. https://www.inegi.org.mx/contenidos/saladeprensa/boletines/2020/EstSociodemo/DefuncionesRegistradas2019.pdf.

[21] Vazquez-Muñoz R., Bogdanchikova N., Huerta-Saquero A. (2020). Beyond the Nanomaterials Approach: Influence of Culture Conditions on the Stability and Antimicrobial Activity of Silver Nanoparticles. ACS Omega.

[22] Uraskulova B.B., Gyusan A.O. (2017). The clinical and bacteriological study of the effectiveness of the application of silver nanoparticle for the treatment of tuberculosis. Vestn. Otorinolaringol..

[23] Romo-Quiñonez C.R., Álvarez-Sánchez A.R., Álvarez-Ruiz P., Chávez-Sánchez M.C., Bogdanchikova N., Pestryakov A., Mejia-Ruiz C.H. (2020). Evaluation of a new Argovit as an antiviral agent included in feed to protect the shrimp Litopenaeus vannamei against white spot syndrome virus infection. PeerJ.

[24] Glotov A.G., Glotova T.I., Sergeev A.A., Belkina T.V., Sergeev A.N. (2004). [Antiviral activity of different drugs in vitro against viruses of bovine infectious rhinotracheitis and bovine diarrhea]. Vopr. Virusol..

[25] Glotov A.G., Glotova T.I., Sergeev A.A., Sergeev A.N. (2004). Study of antiviral activity of different drugs against bovine herpes and pestivirus. Antibiot. I Khimioterapiia = Antibiot. Chemoterapy [Sic].

[26] Juarez-Moreno K., Gonzalez E., Girón-Vazquez N., Chávez-Santoscoy R., Mota-Morales J., Perez-Mozqueda L., Garcia-Garcia M., Pestryakov A., Bogdanchikova N. (2017). Comparison of cytotoxicity and genotoxicity effects of silver nanoparticles on human cervix and breast cancer cell lines. Hum. Exp. Toxicol..

[27] Valenzuela-Salas L.M., Girón-Vázquez N.G., García-Ramos J.C., Torres-Bugarín O., Gómez C., Pestryakov A., Villarreal-Gómez L.J., Toledano-Magaña Y., Bogdanchikova N. (2019). Antiproliferative and Antitumour Effect of Nongenotoxic Silver Nanoparticles on Melanoma Models. Oxid. Med. Cell. Longev..

[28] Fuentes-Valencia M.A., Fajer-Ávila E.J., Chávez-Sánchez M.C., Martínez-Palacios C.A., Martínez-Chávez C.C., Junqueira-Machado G., Lara H.H., Raggi L., Gómez-Gil B., Pestryakov A.A. (2020). Silver nanoparticles are lethal to the ciliate model Tetrahymena and safe to the pike silverside Chirostoma estor. Exp. Parasitol..

[29] Bravo-Guerra C., Cáceres-Martínez J., Vásquez-Yeomans R., Pestryakov A., Bogdanchikova N. (2020). Lethal effects of silver nanoparticles on Perkinsus marinus, a protozoan oyster parasite. J. Invertebr. Pathol..

[30] Pimentel-Acosta C.A., Morales-Serna F.N., Chávez-Sánchez M.C., Lara H.H., Pestryakov A., Bogdanchikova N., Fajer-Ávila E.J. (2019). Efficacy of silver nanoparticles against the adults and eggs of monogenean parasites of fish. Parasitol. Res..

[31] Ochoa-Meza A.R., Álvarez-Sánchez A.R., Romo-Quiñonez C.R., Barraza A., Magallón-Barajas F.J., Chávez-Sánchez A., García-Ramos J.C., Toledano-Magaña Y., Bogdanchikova N., Pestryakov A. (2019). Silver nanoparticles enhance survival of white spot syndrome virus infected Penaeus vannamei shrimps by activation of its immunological system. Fish Shellfish Immunol..

[32] Castro-Gamboa S., Garcia-Garcia M.R., Piñon-Zarate G., Rojas-Lemus M., Jarquin-Yañez K., Angel Herrera-Enriquez M., Fortoul T.I., Toledano-Magaña Y., Garcia-Iglesias T., Pestryakov A. (2019). Toxicity of silver nanoparticles in mouse bone marrow-derived dendritic cells: Implications for phenotype. J. Immunotoxicol..

[33] Kitada N., Takara K., Minegaki T., Itoh C., Tsujimoto M., Sakaeda T., Yokoyama T. (2008). Factors affecting sensitivity to antitumor platinum derivatives of human colorectal tumor cell lines. Cancer Chemother. Pharmacol..

[34] Fenech M. (2000). The in vitro micronucleus technique. Mutat. Res.-Fundam. Mol. Mech. Mutagen..

[35] (2002). Test No. 420: Acute Oral Toxicity-Fixed Dose Procedure.

[36] Toledano-Magaña Y., Meléndrez-Luévano R., Navarro-Olivarria M., García-Ramos J.C., Flores-Alamo M., Ortiz-Frade L., Ruiz-Azuara L., Cabrera-Vivas B.M. (2014). Synthesis, characterization and evaluation of the substituent effect on the amoebicide activity of new hydrazone derivatives. Medchemcomm.

[37] Hernández-Ayala L.F., Toledano-Magaña Y., Ortiz-Frade L., Flores-Alamo M., Galindo-Murillo R., Reina M., García-Ramos J.C., Ruiz-Azuara L. (2020). Heteroleptic NiII complexes: Synthesis, structural characterization, computational studies and amoebicidal activity evaluation. J. Inorg. Biochem..

[38] Pang C., Brunelli A., Zhu C., Hristozov D., Liu Y., Semenzin E., Wang W., Tao W., Liang J., Marcomini A. (2016). Demonstrating approaches to chemically modify the surface of Ag nanoparticles in order to influence their cytotoxicity and biodistribution after single dose acute intravenous administration. Nanotoxicology.

[39] Recordati C., De Maglie M., Bianchessi S., Argentiere S., Cella C., Mattiello S., Cubadda F., Aureli F., D’Amato M., Raggi A. (2016). Tissue distribution and acute toxicity of silver after single intravenous administration in mice: Nano-Specific and size-Dependent effects. Part. Fibre Toxicol..

[40] (2019). Globally Harmonized System of Classification and Labelling of Chemicals (GHS).

[41] Sánchez-López E., Gomes D., Esteruelas G., Bonilla L., Lopez-Machado A.L., Galindo R., Cano A., Espina M., Ettcheto M., Camins A. (2020). Metal-Based nanoparticles as antimicrobial agents: An overview. Nanomaterials.

[42] Krishnan P.D., Banas D., Durai R.D., Kabanov D., Hosnedlova B., Kepinska M., Fernandez C., Ruttkay-Nedecky B., Nguyen H.V., Farid A. (2020). Silver nanomaterials for wound dressing applications. Pharmaceutics.

[43] Zielińska A., Costa B., Ferreira M.V., Miguéis D., Louros J.M.S., Durazzo A., Lucarini M., Eder P., Chaud M.V., Morsink M. (2020). Nanotoxicology and nanosafety: Safety-By-Design and testing at a glance. Int. J. Environ. Res. Public Health.

[44] Yan J., Zhou T., Cunningham C.K., Chen T., Jones M.Y., Abbas M., Li Y., Mei N., Guo X., Moore M.M. (2016). Size- and coating-Dependent cytotoxicity and genotoxicity of silver nanoparticles evaluated using in vitro standard assays. Nanotoxicology.

[45] Nallanthighal S., Chan C., Bharali D.J., Mousa S.A., Vásquez E., Reliene R. (2017). Particle coatings but not silver ions mediate genotoxicity of ingested silver nanoparticles in a mouse model. NanoImpact.

[46] Wu M., Guo H., Liu L., Liu Y., Xie L. (2019). Size-dependent cellular uptake and localization profiles of silver nanoparticles. Int. J. Nanomed..

[47] Akter M., Sikder M.T., Rahman M.M., Ullah A.K.M.A., Hossain K.F.B., Banik S., Hosokawa T., Saito T., Kurasaki M. (2018). A systematic review on silver nanoparticles-induced cytotoxicity: Physicochemical properties and perspectives. J. Adv. Res..

[48] Vecchio G., Fenech M., Pompa P.P., Voelcker N.H. (2014). Lab-On-A-Chip-Based high-Throughput screening of the genotoxicity of engineered nanomaterials. Small.

[49] Ahlberg S., Antonopulos A., Diendorf J., Dringen R., Epple M., Flöck R., Goedecke W., Graf C., Haberl N., Helmlinger J. (2014). PVP-Coated, negatively charged silver nanoparticles: A multi-Center study of their physicochemical characteristics, cell culture and in vivo experiments. Beilstein J. Nanotechnol..

[50] Foldbjerg R., Olesen P., Hougaard M., Anh D., Jürgen H., Autrup H., Dang D.A., Hoffmann H.J., Autrup H. (2009). PVP-Coated silver nanoparticles and silver ions induce reactive oxygen species, apoptosis and necrosis in THP-1 monocytes. Toxicol. Lett..

[51] Foldbjerg R., Jiang X., Micləuş T., Chen C., Autrup H., Beer C. (2015). Silver nanoparticles-Wolves in sheep’s clothing?. Toxicol. Res. (Camb).

[52] Li Y., Qin T., Ingle T., Yan J., He W., Yin J.J., Chen T. (2017). Differential genotoxicity mechanisms of silver nanoparticles and silver ions. Arch. Toxicol..

[53] Kim S., Ryu D.Y. (2013). Silver nanoparticle-induced oxidative stress, genotoxicity and apoptosis in cultured cells and animal tissues. J. Appl. Toxicol..

[54] Mao B.H., Tsai J.C., Chen C.W., Yan S.J., Wang Y.J. (2016). Mechanisms of silver nanoparticle-induced toxicity and important role of autophagy. Nanotoxicology.

[55] Joardar N., Guevara-Flores A., Martínez-González J.D.J., Sinha Babu S.P. (2020). Thiol antioxidant thioredoxin reductase: A prospective biochemical crossroads between anticancer and antiparasitic treatments of the modern era. Int. J. Biol. Macromol..

[56] Kirtonia A., Gala K., Fernandes S.G., Pandya G., Pandey A.K., Sethi G., Khattar E., Garg M. (2020). Repurposing of drugs: An attractive pharmacological strategy for cancer therapeutics. Semin. Cancer Biol..

[57] Borrego B., Lorenzo G., Mota-Morales J.D., Almanza-Reyes H., Mateos F., López-Gil E., de la Losa N., Burmistrov V.A., Pestryakov A.N., Brun A. (2016). Potential application of silver nanoparticles to control the infectivity of Rift Valley fever virus in vitro and in vivo. Nanomed. Nanotechnol. Biol. Med..

[58] Bogdanchikova N., Vázquez-Muñoz R., Huerta-Saquero A., Peña-Jasso A., Aguilar-Uzcanga G., Picos-Díaz P.L., Pestryakov A., Burmistrov V.A., Martynyuk O., Luna-Vázquez-Gómez R. (2016). Silver nanoparticles composition for treatment of distemper in dogs. Int. J. Nanotechnol..

[59] Guerra J.D., Sandoval G., Avalos-Borja M., Pestryakov A., Garibo D., Susarrey-Arce A., Bogdanchikova N. (2020). Selective antifungal activity of silver nanoparticles: A comparative study between Candida tropicalis and Saccharomyces boulardii. Colloid Interface Sci. Commun..

[60] D’Arcy M.S. (2019). Cell death: A review of the major forms of apoptosis, necrosis and autophagy. Cell Biol. Int..

[61] Xu Z., Feng Q., Wang M., Zhao H., Lin Y., Zhou S. (2020). Green Biosynthesized Silver Nanoparticles With Aqueous Extracts of Ginkgo Biloba Induce Apoptosis via Mitochondrial Pathway in Cervical Cancer Cells. Front. Oncol..

[62] Cid M.G., Mudry M., Larripa I. (1995). Chromosome damage induced by carboplatin (CBDCA). Toxicol. Lett..

[63] Calvert A.H., Harland S.J., Newell D.R., Siddik Z.H., Jones A.C., Mcelwain T.J., Raju S., Wiltshaw E., Smith I.E., Baker J.M. (1982). Early Clinical Studies with cis-Diammine-1,1-Cyclobutane Dicarboxylate Platinum II. Cancer Chemother. Pharmacol..

[64] Chen X., Wang J., Fu Z., Zhu B., Wang J., Guan S., Hua Z. (2017). Curcumin activates DNA repair pathway in bone marrow to improve carboplatin-Induced myelosuppression. Sci. Rep..

[65] Vijayakumar S., Malaikozhundan B., Saravanakumar K., Durán-Lara E.F., Wang M.H., Vaseeharan B. (2019). Garlic clove extract assisted silver nanoparticle–Antibacterial, antibiofilm, antihelminthic, anti-inflammatory, anticancer and ecotoxicity assessment. J. Photochem. Photobiol. B Biol..

[66] Tripathy S., Rademan S., Matsabisa M.G. (2020). Effects of Silver Nanoparticle from Dicoma anomala Sond. Root Extract on MCF-7 Cancer Cell Line and NF54 Parasite Strain: An In Vitro Study. Biol. Trace Elem. Res..

[67] Huang H., Lai W., Cui M., Liang L., Lin Y., Fang Q., Liu Y., Xie L. (2016). An Evaluation of Blood Compatibility of Silver Nanoparticles. Sci. Rep..

[68] Joksic G., Stasic J., Filipovic J., Sobot A.V., Trtica M., Joksić G., Stašić J., Filipović J., Šobot A.V., Trtica M. (2016). Size of silver nanoparticles determines proliferation ability of human circulating lymphocytes in vitro. Toxicol. Lett..

[69] Shaniba V.S., Aziz A.A., Jayasree P.R., Kumar P.R.M. (2019). Manilkara zapota (L.) P. Royen Leaf Extract Derived Silver Nanoparticles Induce Apoptosis in Human Colorectal Carcinoma Cells Without Affecting Human Lymphocytes or Erythrocytes. Biol. Trace Elem. Res..

[70] Chakraborty B., Pal R., Ali M., Singh L.M., Rahman D.S., Ghosh S.K., Sengupta M. (2016). Immunomodulatory properties of silver nanoparticles contribute to anticancer strategy for murine fibrosarcoma. Cell. Mol. Immunol..

[71] Liao C., Li Y., Tjong S.C. (2019). Bactericidal and cytotoxic properties of silver nanoparticles. Int. J. Mol. Sci..

[72] Everds N.E. (2015). Evaluation of Clinical Pathology Data:Correlating Changes with Other Study Data. Toxicol. Pathol..

[73] Kim J.S., Song K.S., Sung J.H., Ryu H.R., Choi B.G., Cho H.S., Lee J.K., Yu I.J. (2013). Genotoxicity, acute oral and dermal toxicity, eye and dermal irritation and corrosion and skin sensitisation evaluation of silver nanoparticles. Nanotoxicology.

[74] Maneewattanapinyo P., Banlunara W., Thammacharoen C., Ekgasit S., Kaewamatawong T. (2011). An evaluation of acute toxicity of colloidal silver nanoparticles. J. Vet. Med. Sci..

[75] Melnik E.A., Buzulukov Y.P., Demin V.F., Demin V.A., Gmoshinski I.V., Tyshko N.V., Tutelyan V.A. (2013). Transfer of silver nanoparticles through the placenta and breast milk during in vivo experiments on rats. Acta Nat..

